# The effect of myo-inositol on assisted reproductive technology outcomes in women with polycystic ovarian syndrome: A systematic review and meta-analysis of randomized clinical trial studies

**DOI:** 10.18502/ijrm.v23i5.19260

**Published:** 2025-07-29

**Authors:** Azadeh Akbari Sene, Maryam Saeedzarandi, Maryam Yazdizadeh, Seyede Razieh Ghaffari, Fatemehsadat Amjadi, Zahra Zandieh, Yousef Moradi

**Affiliations:** ^1^Shahid Akbar Abadi Clinical Research Development Unit (ShACRDU), School of Medicine, Iran University of Medical Sciences (IUMS), Tehran, Iran.; ^2^Reproductive Sciences and Technology Research Center, Department of Anatomy, Iran University of Medical Sciences, Tehran, Iran.; ^3^Department of Obstetrics and Gynecology, Medical School, Birjand University of Medical Sciences, Birjand, Iran.; ^4^Social Determinants of Health Research Center, Research Institute for Health Development, Kurdistan University of Medical Sciences, Sanandaj, Iran.

**Keywords:** D-chiro-inositol-galactosamine, Inositol, Assisted reproductive techniques, Polycystic ovarian syndrome, Evidence synthesis.

## Abstract

**Background:**

Conflicting evidence from clinical trials on the effects of myo-inositol and D-chiro-inositol on assisted reproductive technology (ART) outcomes in women with polycystic ovarian syndrome (PCOS) necessitates a systematic review and meta-analysis.

**Objective:**

To evaluate the effect of myo-inositol and D-chiro-inositol on ART outcomes in women with PCOS.

**Materials and Methods:**

A comprehensive search was conducted in PubMed, Scopus, Web of Science, EMBASE, ClinicalTrials.gov, and the Cochrane Library for studies published from January 2000–2023. Statistical analyses were performed using STATA version 17.

**Results:**

17 intervention studies were included. myo-inositol/D-chiro-inositol supplementation significantly increased the clinical pregnancy rate (RR: 1.64, 95% CI: 1.25–2.15; I^2^ = 13.5%; p = 0.32) and top-grade embryos (RR: 1.12, 95% CI: 1.02–1.23; I^2^ = 85.43%; p = 0.0001). However, it was associated with reductions in antral follicle count (WMD: -0.78, 95% CI: -1.07 to -0.49; I^2^ = 97.37%; p = 0.001) and anti-Mullerian hormone levels (WMD: -0.46, 95% CI: -0.67 to -0.25; I^2^ = 91.95%; p = 0.001).

**Conclusion:**

This meta-analysis provides reliable evidence on the effects of myo-inositol/D-chiro-inositol on fertility and ovarian function in women with PCOS undergoing ART.

## 1. Introduction

Polycystic ovarian syndrome (PCOS) is the prevailing endocrine disorder among women of reproductive age, impacting 5–10% of this demographic (1, 2). PCOS is characterized by having 2 of these conditions: excess androgen, dysfunction in ovulation, or abnormal ovarian morphology. Ultrasound is necessary when there is no androgen excess or ovulatory dysfunction. All women should be tested for conditions such as thyroid disease (measured by thyroid-stimulating hormone levels), hyperprolactinemia (assessed through prolactin levels), and non-classic congenital adrenal hyperplasia (evaluated via serum 17-OHP), as determined by clinical judgment (3).

Obesity and insulin resistance are the main factors in the pathophysiology of this disease in women affected by this syndrome (4). It is the primary reason for infertility, following issues with ovulation. Inositol, a B-complex vitamin with antioxidant properties, along with its stereoisomers myo-inositol and D-chiro-inositol, have been researched as a successful therapy for PCOS. The use of inositol in PCOS has been demonstrated to enhance metabolic and hormonal aspects, as well as ovarian function and response to assisted reproductive technology (ART) (5). Myo-inositol could work by different mechanisms besides increasing insulin sensitivity, such as facilitating the nuclear maturation of germinal vesicle oocytes (5, 6). Research indicated that the utilization of myo-inositol may impact fertility by improving the quality of eggs and embryos in individuals with infertility issues. While numerous studies have looked into how myo-inositol affects fertility in individuals with PCOS, the findings have been highly inconsistent (7, 8). However, only a small number of systematic reviews and meta-analyses have been released in this area, and it has been at least 3–4 yr since they were published, emphasizing the necessity of updating these findings (9–13). So, the current research objective was to carry out a detailed evaluation and meta-analysis of the main clinical trial investigations to establish the impact of myo-inositol on ART results in women with PCOS. The current meta-analysis findings could have a significant impact, providing new information and results that can inform clinical decision-making and guide updates to care and treatment protocols.

## 2. Materials and Methods

This study is a systematic review and meta-analysis that consists of 6 key steps: search syntax and strategy, screening, selection, data extraction, quality assessment, and meta-analysis. The research followed the guidelines set by the Preferred Reporting Items for Systematic Reviews and Meta-Analyses (PRISMA) for conducting and reporting its findings (14). The study protocol was not recorded due to time constraints.

### Eligibility criteria

A manual search was conducted to review the relevant article resources to finalize the search and selection process. After collecting all relevant articles, the findings were input into EndNote software version 9. In the following phase, replicate research was eliminated based on the title, author, and publication year of the articles. Next, the research was evaluated by examining the title, abstract, and complete text. To conduct the screening process, the specific criteria for selecting studies were thoroughly reviewed. Meta-analysis included studies structured with population, intervention, comparison, outcome, type of studies (Table I).

Studies without this framework were not included in the current meta-analysis. 2 authors independently carried out all stages of the search strategy and article screening process.

### Search strategy and screening

The meta-analysis results offer trustworthy data on how myo-inositol/D-chiro-inositol affects fertility and ovarian function in women with PCOS during ART cycles. The search included databases like PubMed (Medline), Scopus, Web of Science, EMBASE, ClinicalTrials.gov, and Cochrane Library. The search covered the period from January 2000 and August 2022 and was updated through January 2023. The key terms examined in the research were “Assisted Reproductive Technology", “In vitro fertilization", “Polycystic Ovary Syndrome", “Myo-inositol", “Inofolic", “Infertility", “Embryo Quality", “Mature Oocyte", “Oocyte Quality", “Live Birth Rate", “Ongoing Pregnancy". Thesauruses, Emtree, and MeSH, were used to discover synonyms for a comprehensive search. Once the synonyms were selected, search syntaxes for each database were organized and developed with the use of AND and OR operators. Search syntaxes were reported in table I.

**Table 1:** Criteria for inclusion of studies in the present meta-analysis

**Population (P)**	**Intervention (I)**	**Comparison (C)**	**Outcome (O)**	**Type of study (T)**
The target population in this meta-analysis was women with PCOS in ART cycles	The desired intervention in the present meta-analysis was receiving myo-inositol/D-chiro inositol with different doses in women with PCOS	The comparison group included other drugs or placebo	The intended outcomes included ART outcomes (the clinical pregnancy rate, live birth rate, number of top-grade embryos, OHSS rate, number of oocytes retrieved, and number of metaphase II oocytes retrieved) and ovarian reserve test (AMH, AFC)	All clinical trial studies
Search strategy syntaxes PubMed: (“Myo-Inositol"(15) OR “D-Chiro-Inositol"(15) OR Myoinositol OR “D-chiro-inositol") AND (“Polycystic Ovary Syndrome"(15) OR “PCOS"[Title/Abstract]) AND (“Assisted Reproductive Technology"(15) OR “In Vitro Fertilization"(15) OR “ART"[Title/Abstract] OR “IVF"[Title/Abstract]) AND (“Fertility"(15) OR “Ovarian Function"[Title/Abstract] OR “Embryo Quality"[Title/Abstract] OR “Mature Oocyte"[Title/Abstract] OR “Oocyte Quality"[Title/Abstract] OR “Live Birth Rate"[Title/Abstract] OR “Ongoing Pregnancy"[Title/Abstract]) (n = 19) Scopus: TITLE-ABS-KEY((Myo-Inositol OR “D-Chiro-Inositol" OR Myoinositol OR “D-chiro-inositol") AND (Polycystic Ovary Syndrome OR PCOS) AND (Assisted Reproductive Technology OR In Vitro Fertilization OR ART OR IVF) AND (Fertility OR “Ovarian Function" OR “Embryo Quality" OR “Mature Oocyte" OR “Oocyte Quality" OR “Live Birth Rate" OR “Ongoing Pregnancy")) (n = 570) Web of sciences: TS=((Myo-Inositol OR “D-Chiro-Inositol" OR Myoinositol OR “D-chiro-inositol") AND (Polycystic Ovary Syndrome OR PCOS) AND (Assisted Reproductive Technology OR In Vitro Fertilization OR ART OR IVF) AND (Fertility OR “Ovarian Function" OR “Embryo Quality" OR “Mature Oocyte" OR “Oocyte Quality" OR “Live Birth Rate" OR “Ongoing Pregnancy")) (n = 210)				
ART: Assisted reproductive technology, PCOS: Polycystic ovary syndrome, AMH: Anti-Mullerian hormone, AFC: Antral follicle count, OHSS: Ovarian hyperstimulation syndrome, IVF: In vitro fertilization				

### Data extraction

Currently, important data components were obtained, such as authors, publication years, countries, sample sizes, participant ages, body mass index (BMI) values, studied populations, interventions, follow-up durations, and meta-analysis outcomes. The results included rates of clinical pregnancy, rates of live births, numbers of high-quality embryos, cases of ovarian hyperstimulation syndrome (OHSS), quantity of retrieved oocytes, levels of AMH, counts of antral follicles, and numbers of retrieved metaphase II oocytes. 2 researchers independently conducted the data extraction process. Any inconsistencies were handled and solved with the help of a third party.

### Quality assessment

The Cochrane risk of bias assessment tool was used to assess the study design, sampling methods, and measurement quality. Specific aspects for the cross-over design in 2 of the studies were addressed following the recommendations in the Cochrane handbook for systematic reviews of interventions. 2 researchers independently evaluated the potential for bias in each trial using Revman 5.3 software. If there were any differences, they were settled by mutual understanding, and if a common decision could not be reached, a third researcher was brought in to resolve the matter.

### Statistical Analysis

The desired effect size in this study included several indexes. The first index was the risk ratio (RR), whose logarithm and standard deviation of the logarithm were calculated, and the pooled RR was reported. The second index was the weighted mean difference (WMD) calculated and reported for the studies that reported the mean of outcomes after the intervention in the intervention and placebo groups. The WMD at the third index was calculated for studies that showed the WMD of outcomes after intervention in both the intervention and placebo groups. The diversity of studies was assessed by employing I^2^ and Cochrane's Q test. As per Cochrane's criteria, 0–25% suggests the absence of heterogeneity, 25–50% signifies mild heterogeneity, 50–75% indicates high yet reasonable heterogeneity, and 75–100% denotes high and unacceptable heterogeneity. Egger's test was employed to assess publication bias. The analysis was conducted with STATA 17.0, and significance was determined at a p < 0.05.

## 3. Results

**Figure 1:**
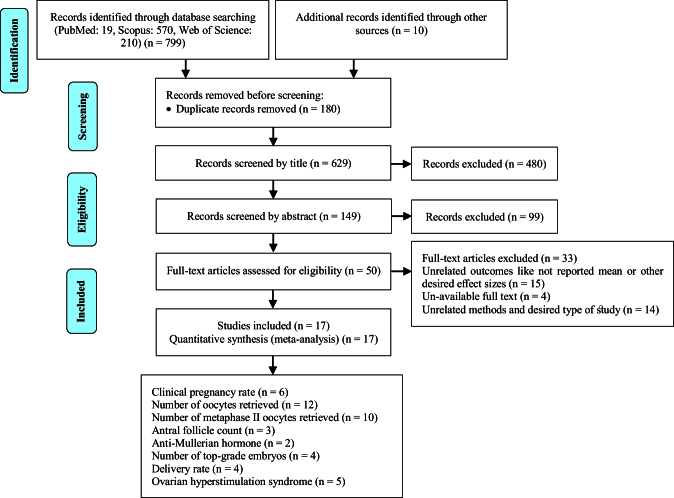
Flow diagram.

In this meta-analysis, a total of 17 interventional studies (randomized clinical trials, non-randomized studies, and before-and-after designs) were included after a thorough search in various databases and manual scanning of the references of the selected articles. Out of the various databases surveyed, a total of 809 studies were identified. After removing 180 duplicates, 629 articles passed on to the title screening stage, where 480 records were excluded based on relevance. Of these, 149 remaining articles were subjected to abstract screening, wherein 99 studies were excluded. Full-text screening of 50 articles was conducted; among these, 33 articles were excluded for the following reasons: unrelated outcomes, which included studies with missing mean values and other desired effect size estimation (n = 15); unavailability of full text (n = 4), and unrelated methods or studies with designs not in response to the set inclusion criteria (n = 14) (Figure 1). Among the selected studies, 6 focused on clinical pregnancy rates, while the number of oocytes retrieved was reported in 12 studies. 10 studies evaluated the number of metaphase II oocytes retrieved. AMH and AFC levels were assessed in 3 studies. 4 studies examined the number of top-grade embryos, and delivery rates were similarly reported in 4 studies. Additionally, 5 studies documented cycle cancellations due to the risk of OHSS (16–18). The majority of studies were conducted in Italy, accounting for 13 of the included studies, while 2 studies each were conducted in Iran and Turkey, and one study each in Germany and Georgia. Across all studies, the population consisted of women with PCOS undergoing intracytoplasmic sperm injection (ICSI) or in vitro fertilization (IVF). Most studies compared intervention groups to either placebo or control groups receiving standard treatments such as folic acid (17–22). The follow-up duration post-treatment was generally more than 12 wk. Notably, 2 Italian studies adopted a pre- and post-intervention design. In one study, the intervention involved administering 800 mg of alpha-lipoic acid combined with myo-inositol, while in the other, D-chiro-inositol was used (16, 18, 21, 23). Results from these studies primarily reported mean differences or post-intervention means for outcomes such as AFC and AMH, as detailed in tables II and III.

### Quantitative results

#### AFC (3 studies)

**Figure 2:**
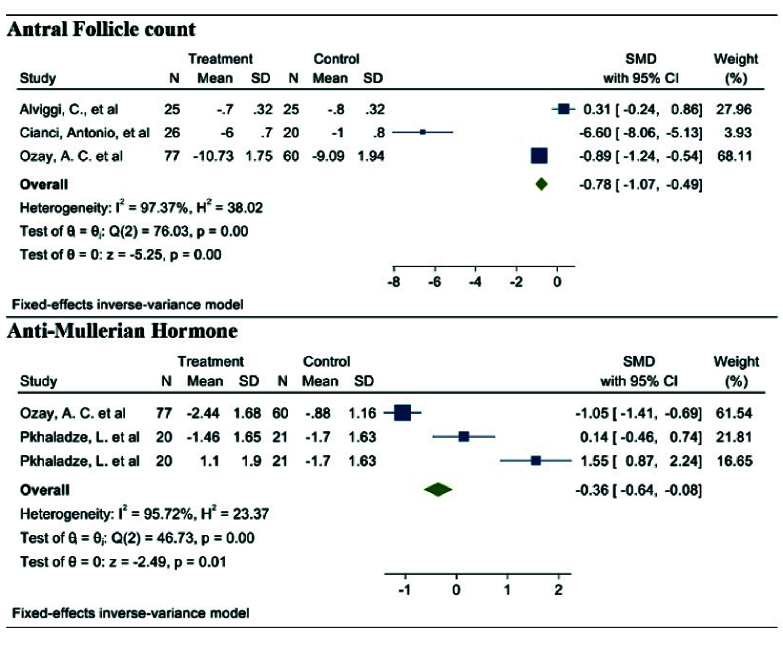
Forest plot of the effect of Myo-inositol/Di-chiro-inositol on AFC and AMH in women with PCOS undergoing IVF/ICSI.

After combining these studies, the WMD was -0.78 follicles (95% CI: -1.07, -0.49; I^2^ = 97.37%, p = 0.001) (Figure 2). This suggests that, in the group consuming myo-inositol/D-chiro-inositol, the mean AFC was 0.78 follicles lower compared to the placebo group, and the difference was statistically significant. Due to the small number of studies (n = 3), analyses related to heterogeneity, publication bias, and meta-regression were not conducted.

#### AMH (2 studies)

After combining these studies, the WMD was -0.36 (95% CI: -0.64, -0.08; I^2^ = 91.95%, p = 0.001) (Figure 2). Thus, in the group consuming myo-inositol/D-chiro-inositol, the mean AMH was 0.36 lower than in the placebo groups and was statistically significant. Due to the small number of studies (n = 3), analyses related to heterogeneity, publication bias, and meta-regression were not performed.

#### Number of oocytes retrieved (12 studies)

Out of the 17 studies that were chosen, 12 of them conducted a comparison of this particular outcome between the 2 groups. In this research, the average and standard deviation of retrieved oocytes were documented in both groups taking myo-inositol/D-chiro-inositol and placebo. The effect size that was aimed for in this meta-analysis was WMD.

**Figure 3:**
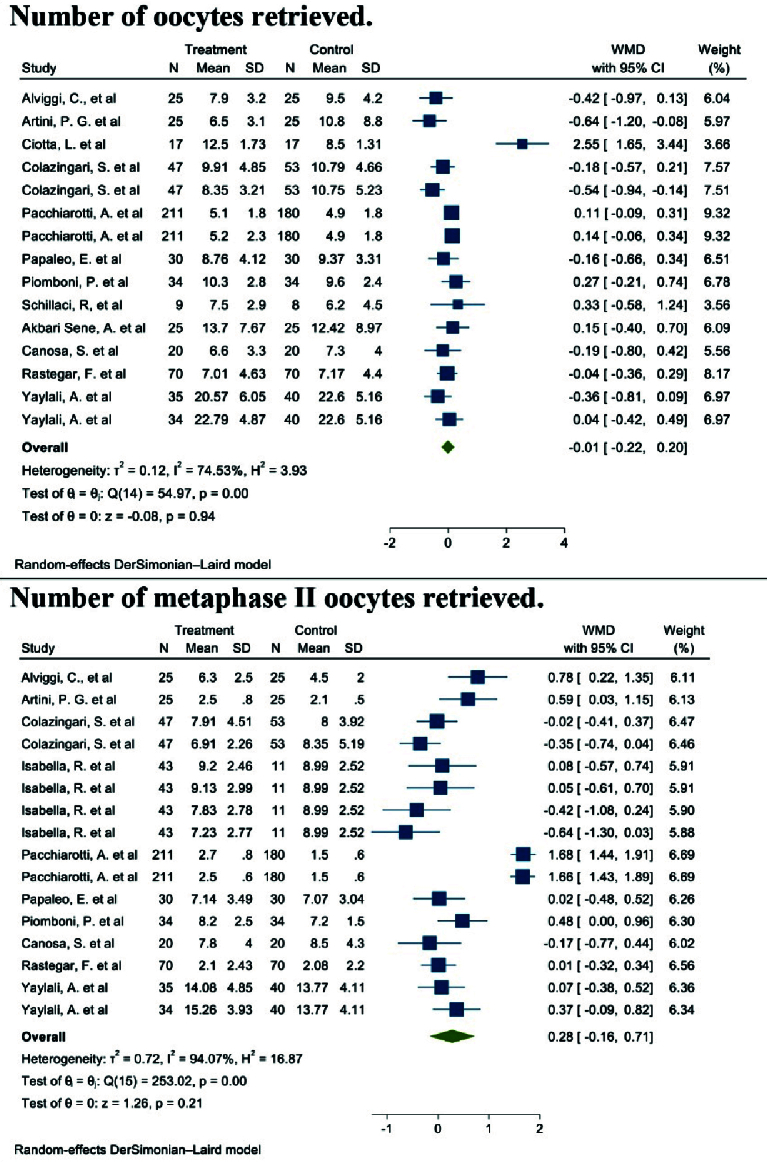
Forest plot of the effect of Myo-inositol/Di-chiro-inositol on number of oocytes retrieved and number of metaphase II oocytes retrieved in women with PCOS undergoing IVF/ICSI.

The combined studies showed a WMD of -0.01 (WMD: -0.01; 95% CI: -0.22, 0.20; I square: 74.53%, p = 0.001) (Figure 3). Hence, in the myo-inositol/D-chiro-inositol group, the average amount of oocytes collected was slightly lower by -0.01 compared to the placebo group, with no statistical significance (Figure 3).

**Figure 4:**
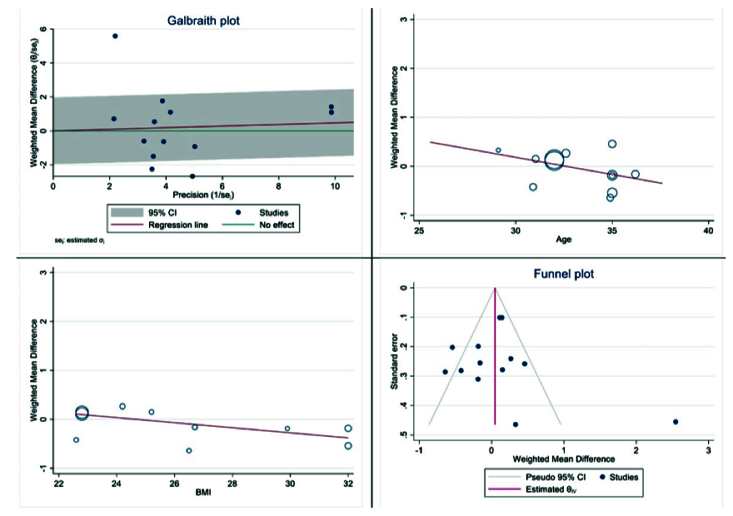
Meta regression, heterogeneity, and funnel plots on the effect of myo-inositol/Di-chiro-inositol on number of oocytes retrieved in women with PCOS undergoing IVF/ICSI.

Figure 4 displays the outcomes of diversity, meta-regression using women's BMI and age who have polycystic ovary syndrome, and the publication bias graph. The findings indicated that the impact of myo-inositol/D-chiro-inositol on the number of collected oocytes diminished as individuals grew older (B: -0.069; SE: 0.048; p = 0.183; 95% CI: -0.178, -0.039). Additionally, as the BMI rose, a decrease was observed in the impact of this medication on the number of retrieved oocytes (B: -0.051; SE: 0.018; p = 0.024; 95% CI: -0.094, -0.008), which was found to be statistically significant (Figure 4). Funnel plot and Egger's test were used to assess publication bias, with results indicating its absence in the analysis of the impact of myo-inositol/D-chiro-inositol on oocyte retrieval in women with PCOS (B: 0.30; SE: 0.60; p = 0.620) (Figure 4).

#### Number of metaphase II oocytes retrieved (10 studies)

After combining these studies, the WMD was 0.28 (WMD: 0.28; 95% CI: -0.16, 0.71; I^2^: 94.07%, p: 0.001) (Figure 3). This means in the group consuming myo-inositol/D-chiro-inositol, the mean number of metaphase II oocytes retrieved was 0.28 higher than the placebo group, and it was statistically significant (Figure 3).

**Figure 5:**
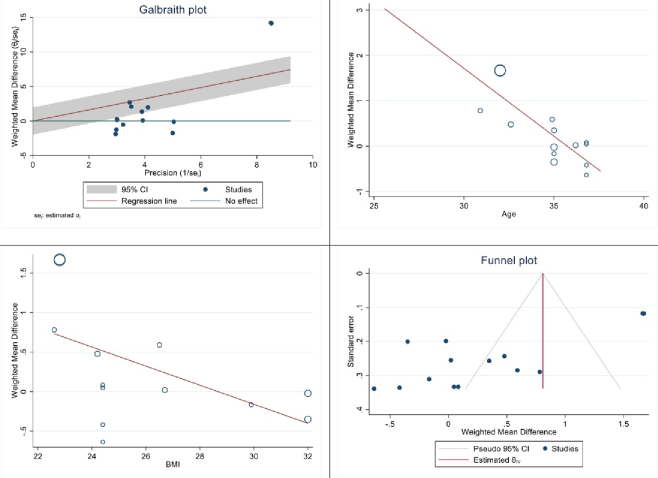
Meta regression, heterogeneity, and funnel plots on the effect of Myo-inositol/Di-chiro-inositol on number of metaphase II oocytes retrieved in women with PCOS undergoing IVF/ICSI.

The results of heterogeneity, meta-regression based on the BMI and age of women with PCOS and the publication bias diagram have been reported in figure 5, the results of which showed with increasing age, the effect of myo-inositol/D-chiro-inositol on the number of metaphase II oocytes retrieved decreased (B: -0.298; SE: 0.064; p: 0.001; 95% CI: -0.439, -0.157). Also, with increasing the BMI, the effect of this drug on the number of metaphase II oocytes retrieved decreased (B: -0.120; SE: 0.057; p = 0.024; 95% CI: -0.24, -0.004), which was not statistically significant (Figure 5). Publication bias was assessed using a Funnel plot and Egger's test. The test results showed that it occurred in the analysis of the effect of myo-inositol/D-chiro-inositol on the number of metaphase II oocytes retrieved in women with PCOS (B: -8.69; SE: 0.69; p = 0.0001) (Figure 5).

#### OHSS rate (5 studies)

**Figure 6:**
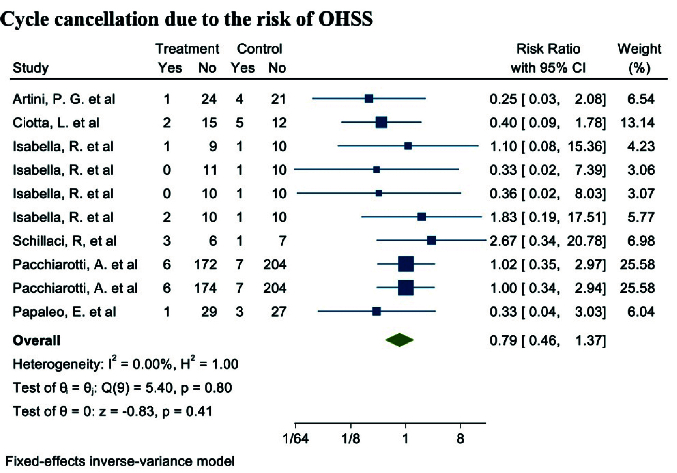
Forest plot the effect of Myo-inositol/Di-chiro-inositol on OHSS rate in women with PCOS undergoing IVF/ICSI.

Out of the 17 selected studies, 5 investigated this outcome in both target groups. The study with the lowest RR reported was 0.25 (95% CI: 0.03; 2.08), while the study with the highest RR observed was 2.67 (95% CI: 0.34; 20.78) (19, 23). After combining the results from these studies, the overall RR was 0.79, indicating that the OHSS rate in the group consuming myo-inositol/D-chiro-inositol was 16% lower compared to the placebo group (RR: 0.79, 95% CI: 0.46; 1.37, I^2^: 0.00%, p = 0.78) (Figure 6).

**Figure 7:**
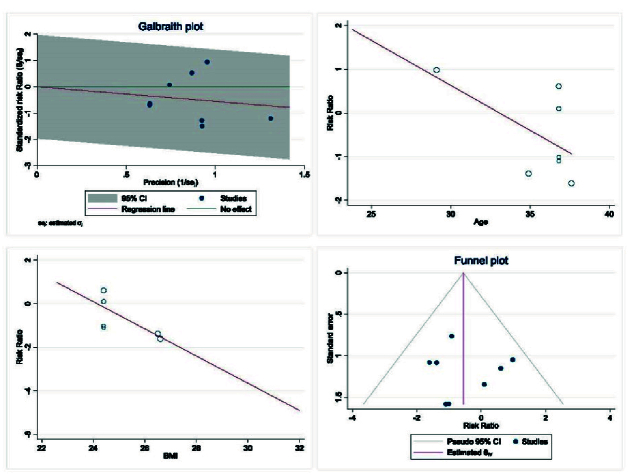
Meta regression, heterogeneity, and funnel plots on the effect of Myo-inositol/Di-chiro-inositol on OHSS rate in women with PCOS undergoing IVF/ICSI.

The results of heterogeneity, meta-regression based on the BMI and age of women with PCOS, and the publication bias diagram have been reported in figure 7. The results showed that the effect of myo-inositol/D-chiro-inositol on cycle cancellation due to the risk of OHSS decreased with age (B: -0.205; SE: 0.150; p = 0.229; 95% CI: -0.591, 0.180). Also, with increasing the BMI, the effect of this drug on cycle cancellation decreased due to the risk of OHSS (B: 0.624; SE: 0.478; p = 0.262; 95% CI: -1.951, 0.703), but the effect of age and the BMI was not statistically significant in meta-regression analysis (Figure 7). Publication bias was assessed using a funnel plot and Egger's test, the results of which showed that it did not occur in the analysis of the effect of myo-inositol/D-chiro-inositol on OHSS rate in women with PCOS (B: 0.26; SE: 1.55; p = 0.866) (Figure 7).

#### Number of top-grade embryos (4 studies)

**Figure 8:**
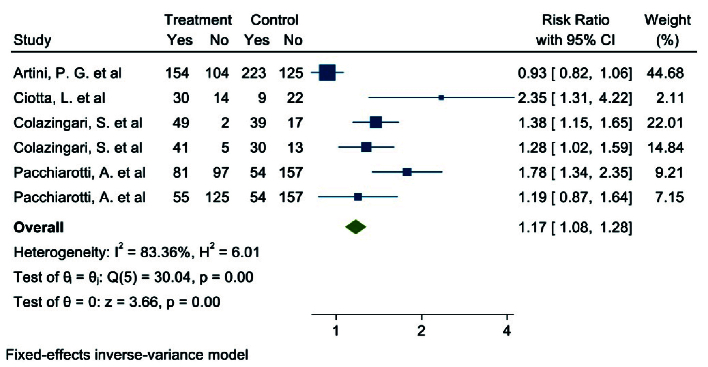
Forest Plot of the effect of Myo-inositol/Di-chiro-inositol on the number of top-grade embryos in women with PCOS undergoing IVF/ICSI.

Out of the 17 selected studies, 4 examined this outcome in both target groups. The study with the lowest RR reported an 0.93 (95% CI: 0.82; 1.06), while the study with the highest RR observed an 2.35 (95% CI: 1.31; 4.22) (19, 21). After combining the results from these studies, the overall RR was 1.17, indicating that in the group consuming myo-inositol/D-chiro-inositol, the number of top-grade embryos was 17% higher compared to the placebo group (RR: 1.17, 95% CI: 1.08; 1.28, I^2^: 83.36%, p = 0.00) (Figure 8). Due to the small number of studies (4), analyses related to heterogeneity, publication bias, and meta-regression were not performed.

#### Clinical pregnancy rate (6 studies)

**Figure 9:**
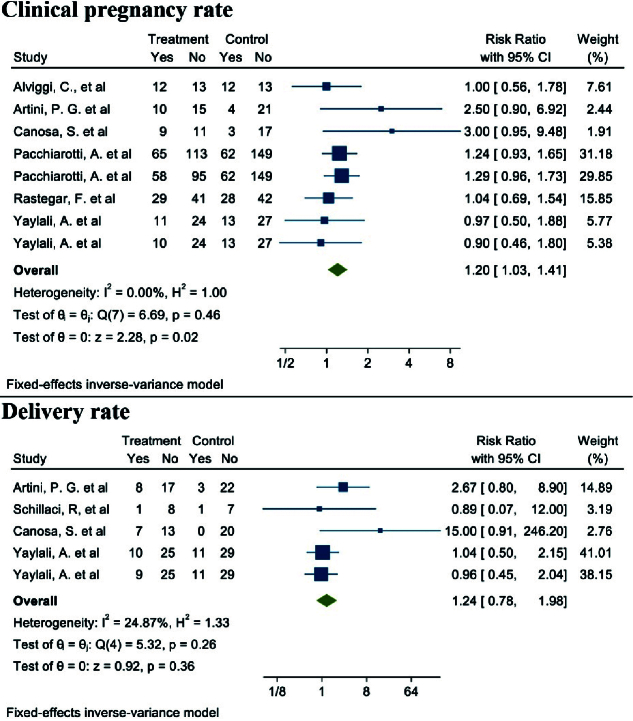
Forest Plot the effect of Myo-inositol/Di-chiro-inositol on clinical pregnancy rate and delivery rate in women with PCOS undergoing IVF/ICSI.

Out of the 17 selected studies, 6 investigated this outcome in both target groups, with the desired effect size represented by the RR. The lowest RR observed was 0.90 (95% CI: 0.46; 1.80), while the highest was 3.00 (95% CI: 0.95; 9.48). After combining these studies, the overall RR was 1.20, indicating that in the group consuming myo-inositol/D-chiro-inositol, the clinical pregnancy rate was 20% higher compared to the placebo group (RR: 1.20, 95% CI: 1.03; 1.41, I^2^: 18.44%, p = 0.27) (Figure 9).

**Figure 10:**
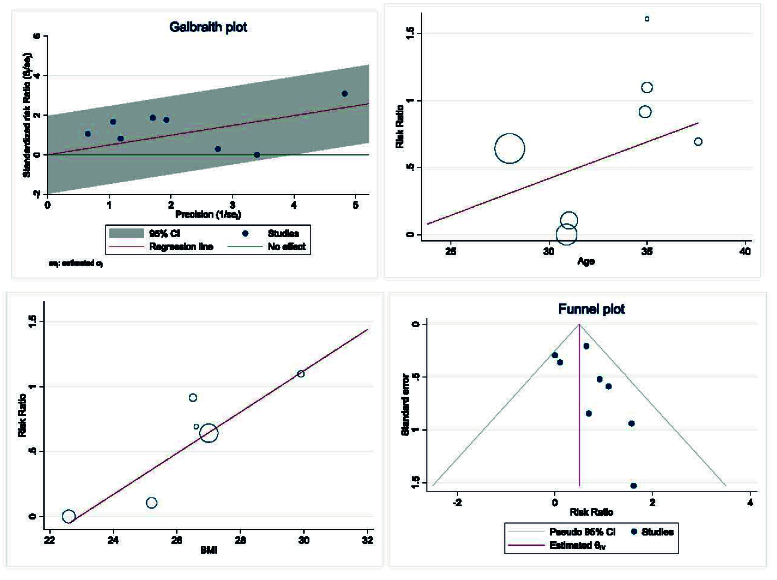
Meta regression, heterogeneity, and funnel plots on the effect of Myo-inositol/Di-chiro-inositol on clinical pregnancy rate in women with PCOS undergoing IVF/ICSI.

Heterogeneity, meta-regression analyses based on BMI and age of women with PCOS, and the publication bias diagram are presented in figure 10. The results showed that the effect of myo-inositol/D-chiro-inositol on clinical pregnancy rates increased with age (B: 0.054; SE: 0.072; p = 0.485; 95% CI: -0.13, 0.24). Additionally, as BMI increased, the effect of the drug on clinical pregnancy rates also increased (B: 0.158; SE: 0.069; p = 0.085; 95% CI: -0.03, 0.35). However, neither age nor BMI had a statistically significant impact on the meta-regression analysis (Figure 10).

Publication bias was evaluated using a funnel plot and Egger's test. The results indicated no evidence of publication bias in the analysis of the effect of myo-inositol/D-chiro-inositol on clinical pregnancy rates in women with PCOS (B: 0.90; SE: 0.69; p = 0.192) (Figure 10).

#### Live birth rate (4 studies)

Out of the 17 selected studies, 4 examined this outcome in both target groups. The lowest RR observed was 0.86 (95% CI: 0.07; 12.00), and the highest RR was 15.00 (95% CI: 0.91; 246.20). After combining these studies, the overall RR was 1.24. Therefore, in the group consuming myo-inositol/D-chiro-inositol, the delivery rate was 1.24 times higher than in the placebo group (RR: 1.24, 95% CI: 0.78; 1.98, I^2^: 34.17%, p = 0.19) (Figure 9). Due to the small number of studies (4), analyses related to heterogeneity, publication bias, and meta-regression were not conducted.

**Table 2:** The characteristics of selected studies related to the effect of Myo-inositol/Di-chiro-inositol on ART outcomes in women with PCOS undergoing IVF/ICSI

** Author, Yr (Ref)**	**Sample size**	**Mean BMI Mean age**	**Study population**	**Type of intervention**	**Duration (wk)**	**Clinical pregnancy rates/ live birth rate**	**Cycle cancellation due to the risk of OHSS**	**Number of top-grade embryos Top-quality embryos**
Alviggi *et al.*, 2016 (24)	Total (50) Intervention (25) Control (25)	30.9 ± 2.4 22.6 ± 2.4	Women with PCOs undergoing IVF/ICSI	Intervention: FT 500 plus, 2 sachets per day for 5 months preceding IVF/ICSI treatment	20	12 (25) NR	NR	NR NR
				Control: received no medication		12 (25) NR	NR	NR NR
Artini *et al.*, 2013 (25)	Total (50) Intervention (25) Control (25)	34.9 ± 1.9 26.5 ± 2.7	Obesity/overweight women with PCOs undergoing IVF/ICSI	Intervention: received 2 gr of myo-inositol and 400 µg of folic acid for 12 wk preceding IVF/ICSI treatment	12	10 (25) 8 (25)	1 (25)	54%
				Control: received 400 µg of folic acid for 12 wk preceding IVF/ICSI treatment		4 (25) 3 (25)	4 (25)	64%
Cianci *et al.*, 2015 (26)	Total (46) Intervention (26) Control (20)	23.8 ± 2.5 28.8 ± 2.7	Women with PCOS, according to the Rotterdam criteria	Intervention: received 1000 mg D-chiro-inositol and 600 mg lipoic	25	NR NR	NR	NR NR
				Control: received no treatment		NR NR	NR	NR NR
Ciotta *et al.*, 2011 (27)	Total (34) Intervention (17) Control (17)	NR NR	Women with PCOS according to the Rotterdam criteria and < 40 yr old	Intervention: received 4 gr of myo-inositol and 400 µg of folic acid for 3 months preceding IVF/ICSI treatment	12	NR NR	2 (17)	30 (44) NR
				Control: received 400 µg of folic acid for 3 months preceding the treatment		NR NR	5 (17)	9 (31) NR
Colazingari *et al.*, 2013 (23)	Total (100) Intervention (47) Control (53)	35 ± 3.1 32 ± 1.7	Women with PCOS, according to the Rotterdam criteria, with a BMI < 28, FSH < 10 IU/l, and a normal uterine cavity	Intervention: received MI 550 mg and D-chiro-inositol 13.8 mg orally twice a day for 12 wk preceding IVF/ICSI treatment	12	NR	NR	≤ 35, 49 (51), > 35, 41 (46) NR
				Control: received D-chiro-inositol 500 mg orally twice a day for 12 wk preceding IVF/ICSI treatment		NR	---	≤ 35, 39 (56), > 35, 30 (43) NR
Isabella *et al.*, 2012 (16)	Total (54) Intervention (43) Control (11)	36.8 ± 1.5 24.4 ± 2.8	Women with PCOS, according to the Rotterdam criteria, between 18 and 35 yr	Intervention (10): received 300 mg of D-chiro-inositol for 8 wk preceding ICSI treatment	24	NR	1 (10)	NR
				Intervention (11): received 600 mg of D-chiro-inositol for 8 wk preceding ICSI treatment		NR	0 (11)	NR
				Intervention (10): received 1200 mg of D-chiro-inositol for 8 wk preceding ICSI treatment		NR	0 (10)	NR
				Intervention (12): received 2400 mg of D-chiro-inositol for 8 wk preceding ICSI treatment		NR	2 (12)	NR
				Control (11): received placebo for 8 wk preceding ICSI treatment		NR	1 (11)	NR
De Cicco *et al.*, 2017 (28)	Total (40)	25.57 ± 5.7 30.03 ± 4.4	Women with PCOS, according to the Rotterdam criteria, between 18 and 35 yr	Participants received ALA (800 mg) and myo-inositol (2000 mg) daily for 6 months	24	NR	NR	NR
				NA				
La Marca *et al.*, 2015 (29)	Total (47)	27.5 ± 6.7 23.0 ± 4.1	Women with PCOS according to the Rotterdam criteria	Participants received 1000-1500 mg of Di-chiro inositol for varying durations	48	NR	NR	NR
				NA				
Lesoine *et al.*, 2016 (30)	Total (29) Intervention (15) Control (14)	36.8 ± 4.3 24.4 ± 2.9	Women with PCOS, according to the Rotterdam criteria, between 18 and 35 yr	Intervention: received 4 gr myo-inositol + 400 µg folic acid for 2 months preceding IVF treatment	8	NR	NR	NR
				Control: received placebo for 2 months preceding IVF/ICSI treatment		NR	NR	NR
Ozay *et al.*, 2016 (31)	Total (137) Intervention (77) Control (60)	22.79 ± 4.1 23.79 ± 4.2	Women with PCOS according to the Rotterdam criteria	Intervention: received 2 gr myo-inositol and 200 µg of folic acid for 12-16 wk before IVF/ICSI treatment	16	NR	NR	NR
				Control: received the COC pill for 3 months preceding IVF/ICSI treatment		NR	NR	NR
Pacchiarotti *et al.*, 2016 (17)	Total (569) Intervention 1 (178) Intervention 2 (180) Control (211)	32.0 ± 3.6 22.8 ± 1.3	Women with PCOS between 27-38 yr, according to the Rotterdam criteria	Intervention 1: 178 participants received 4000 mg of myo-inositol, 400 µg of folic acid, and 3 gr of melatonin	12	65 (178)	6 (178)	45.7%
				Intervention 2: 180 participants received 4000 mg of myo-inositol and 400 µg of folic acid		58 (180)	6 (180)	30.4%
				Control: received 400 µg of folic acid		62 (211)	7 (211)	25.6%
Papaleo *et al.*, 2009 (18)	Total (60) Intervention (30) Control (30)	36.2 ± 2.4 26.7 ± 7.5	Women < 40 yr with PCOS, according to the Rotterdam criteria	Intervention: received 4 gr of myo-inositol and 400 µg of folic acid	---	8 (30)	1 (30)	0.86 ± 0.83%
				Control: received 400 µg of folic acid		7 (30)	3 (30)	0.81 ± 0.83%
Piomboni *et al.*, 2014 (32)	Total (68) Intervention (46) Control (22)	32.6 ± 4.2 24.2 ± 4.6	Women with PCOS, according to the Rotterdam criteria, undergoing IVF/ICSI treatment	Intervention (26): received di-chiro inositol 500 mg bid for 3 months preceding IVF/ICSI treatment	12	NR	NR	5.8 ± 1.3
				Intervention (20): received metformin 850 mg bid for 3 months preceding IVF/ICSI treatment		NR	NR	5.5 ± 2.1
				Control (22): no treatment		NR	NR	4.2 ± 1.1
Pkhaladze *et al.*, 2016 (22)	Total (61) Intervention (40) Control (21)	15.9 ± 3.75 22.7 ± 3.60	Adolescent girls between 13 and 19 yr with PCOS, according to the Rotterdam criteria	Treatment 1: 20 individuals were given monophasic low-dose combined oral contraceptive pills, Yarina (containing drospirenone 3 mg/ethinyl estradiol 30 µg), in a cyclic schedule (3^rd^-5^th^ day of menstruation for 21 days) for 3 months	12	NR	NR	NR
				Intervention 2: 20 participants received 4 gr myo-inositol plus 400 mg of folic acid for 3 months		NR	NR	NR
				Control: 21 patients are being administered a mix of Yarina and Inofolic for 3 months		NR	NR	NR
Schillaci *et al.*, 2012 (33)	Total (17) Intervention (9) Control (8)	29.1 ± 4.2 NR	Women with PCOS, according to the Rotterdam criteria, undergoing IVF/ICSI treatment	Intervention: received 4 gr of myo-inositol and 400 µg of folic acid daily for at least 1-month preceding IVF/ICSI treatment	4	1 (9) NR	3 (9)	NR
				Control: received 400 µg of folic acid daily for at least 1-month preceding IVF/ICSI treatment		2 (8) NR	1 (8)	NR
Akbari Sene *et al.*, 2019 (34)	Total (50) Intervention (25) Control (25)	31.03 ± 4.1 25.26 ± 5.2	Women with PCOS, according to the Rotterdam criteria, undergoing ICSI/ART treatment	Intervention: received a daily dose of 4 gr myo-inositol combined with 400 mg of folic acid	4	NR NR	NR	NR
				Control: received a daily dose of 400 mg of folic acid from 1 month before starting the antagonist cycle until the day of ovum pick-up		NR NR	NR	NR
Canosa *et al.*, 2020 (20)	Total (40) Intervention (20) Control (20)	35.0 ± 5.5 29.9 ± 2.0	Obese or overweight Women with PCOS, according to the Rotterdam criteria, undergoing IVF treatment	Intervention: received 2 gr MI + 800 mg ALA + 400 mg folic acid daily for 3 months before COS	12	9 (20) 7 (20)	NR	38 (84)
				Control: received 400 mg folic acid only daily for 3 months before COS		3 (20) 0 (20)	NR	24 (92)
BMI: Body mass index, OHSS: Ovarian hyperstimulation syndrome, PCOS: Polycystic ovary syndromes, IVF: In vitro fertilization, ICSI: Intracytoplasmic sperm injection, FT 500 Plus: Refers to a specific product that contains myo-inositol in combination with antioxidant activities, NR: Not reported, FSH: Follicle-stimulating hormone, MI: Metaphase II oocytes, ALA: Alpha-lipoic acid, COC: Combined oral contraceptive, ART: Assisted reproductive technology, NA: Not applicable. COS: Controlled ovarian stimulation								

**Table 3:** Results of selected studies of the effect of myo-inositol/di-chiro-inositol on markers of ovarian reserve in women with PCOS undergoing IVF/ICSI

**Author, yr (ref)**			**Result (means ± SD)**			
	**Outcome**		**Group**	**Baseline**	**After**	**Mean change in per group**
Alviggi *et al*., 2016 (24)	AFC		I	13.0 ± 1.2	12.3 ± 1.1	-0.70 ± 0.32
			P	13.5 ± 1.2	12.7 ± 1.1	-0.80 ± 0.32
	Number of oocytes retrieved		I	NR	7.9 ± 3.2	NA
			P	NR	9.5 ± 4.2	NA
	Number of MII oocytes retrieved		I	NR	6.3 ± 2.5	NA
			P	NR	4.5 ± 2.0	NA
Artini *et al*., 2013 (25)	Number of oocytes retrieved		I	NR	6.5 ± 3.1	NA
			P	NR	10.8 ± 8.8	NA
	Number of MII oocytes retrieved		I	NR	2.5 ± 0.8	NA
			P	NR	2.1 ± 0.5	NA
Cianci *et al*., 2015 (26)	AFC		I	16 ± 3	10 ± 2	-6.0 ± 0.70
			P	16 ± 2	15 ± 3	-1.0 ± 0.80
Ciotta *et al*., 2011 (27)	Number of oocytes retrieved		I	NR	26	NA
			p		17	
	Number of MII oocytes retrieved		I	NR	82.24%	NA
			P		66.87%	
Colazingari *et al*., 2013 (23)	Number of oocytes retrieved	≤ 35	I	NR	9.91 ± 4.85	NA
			P	NR	10.79 ± 4.66	
		> 35	I	NR	8.35 ± 3.21	NA
			P	NR	10.75 ± 5.23	
	Number of MII oocytes retrieved	≤ 35	I	NR	7.91 ± 4.51	NA
			P	NR	8.00 ± 3.92	
		> 35	I	NR	6.91 ± 2.26	NA
			P	NR	8.35 ± 5.19	
Isabella *et al*., 2012 (16)	Number of oocytes retrieved		I	NR	9.2 ± 2.46	NA
			I	NR	9.13 ± 2.99	NA
			I	NR	7.83 ± 2.78	NA
			I	NR	7.23 ± 2.77	NA
			P	NR	8.99 ± 2.52	NA
De Cicco *et al*., 2017 (28)	AMH		Before	NR	10.61 ± 7.13	NA
			After	NR	7.50 ± 4.94	
	AFC		Before	NR	41.24 ± 6.12	NA
			After	NR	34.39 ± 5.20	
La Marca *et al*., 2015 (29)	AMH	< 12 months	Before	NR	4.3 ± 3.6	NA
			After	NR	3.1 ± 1.2	
		> 12 months	Before	NR	4.3 ± 3.6	NA
			After	NR	2.9 ± 1.1	
Lesoine *et al*., 2016 (30)	Number of oocytes retrieved		I	NR	---	NA
			P		---	
	Number of MII oocytes retrieved		I	NR	---	
			P		---	
Ozay *et al*., 2016 (31)	AFC		I	32.61 ± 10.50	21.88 ± 11.23	-10.73 ± 1.75
			P	30.90 ± 10.27	21.81 ± 10.99	-9.09 ± 1.94
	AMH		I	11.51 ± 11.50	9.07 ± 9.32	-2.44 ± 1.68
			P	9.39 ± 6.60	8.51 ± 6.20	-0.88 ± 1.16
Pacchiarotti *et al*., 2016 (17)	Number of oocytes retrieved		I1	NR	5.1 ± 1.8	NA
			I2	NR	5.2 ± 2.3	
			P	NR	4.9 ± 1.8	
	Number of MII oocytes retrieved		I1	NR	2.7 ± 0.8	NA
			I2	NR	2.5 ± 0.6	
			P	NR	1.5 ± 0.6	
Papaleo *et al*., 2009 (18)	Number of oocytes retrieved		I	NR	8.76 ± 4.12	NA
			P	NR	9.37 ± 3.31	
	Number of MII oocytes retrieved		I	NR	7.14 ± 3.49	NA
			P	NR	7.07 ± 3.04	
Piomboni *et al*., 2014 (32)	Number of oocytes retrieved		I1	NR	10.3 ± 2.8	NA
			I2	NR	9.8 ± 3.1	
			P	NR	9.6 ± 2.4	
	Number of MII oocytes retrieved		I1	NR	8.5 ± 2.5	NA
			I2	NR	8.8 ± 2.2	
			P	NR	7.2 ± 1.5	
Pkhaladze *et al*., 2016 (22)	AMH		I1	11.72 ± 5.8	10.26 ± 4.6	-1.46 ± 1.65
			I2	11.5 ± 5.8	12.6 ± 6.25	1.10 ± 1.90
			P	12.01 ± 5.6	10.4 ± 4.7	-1.70 ± 1.63
Schillaci *et al*., 2012 (33)	Number of oocytes retrieved		I	NR	7.5 ± 2.9	NA
			P	NR	6.2 ± 4.5	
Akbari Sene *et al*., 2019 (34)	Number of oocytes retrieved		I	NR	13.8 ± 7.67	NA
			P	NR	12.4211 ± 8.97	
	Number of MII oocytes retrieved		I	NR	78.67 (76.01, 81.33) (95% CI)	NA
			P	NR	58.28 (53.97, 62.58) (95% CI)	
Canosa *et al*., 2020 (20)	Number of MII oocytes retrieved		I	NR	6.6 ± 3.3	NA
			P	NR	7.3 ± 4.0	
	Number of oocytes retrieved		I	NR	7.8 ± 4.0	NA
			P	NR	8.5 ± 4.3	
PCOS: Polycystic ovary syndrome, AFC: Antral follicle count, AMH: Anti-Mullerian hormone, MII: Metaphase II, I: Intervention group, P: Placebo group, NR: Not reported, NA: Not applicable, IVF: In vitro fertilization, ICSI: Intracytoplasmic sperm injection						

## 4. Discussion

Women with PCOS face increased pregnancy complications and often require fertility support. Myo-inositol, a common supplement, is thought to improve outcomes in ART. This meta-analysis evaluated its effect in women with PCOS and found that combining myo-inositol with D-chiro-inositol increased clinical pregnancy rates by 20% compared to placebo. The association was statistically significant with low heterogeneity ( ∼ 18%), indicating consistent results across studies with sufficient sample sizes. While promising, further research is needed to assess long-term outcomes such as live birth rates, potential side effects, and to standardize dosage and treatment protocols. Future studies should also consider the influence of BMI and age on treatment efficacy. In contrast, the estimated confidence interval was equal to 1.03–1.41, indicating the high reliability of the result due to its narrowness. According to the confidence interval, in at least 3% and at most 41% of women with PCOS, if myo-inositol were taken, the clinical pregnancy rate would increase compared to the placebo group or other treatments. Still, this effect was not statistically significant for the live birth rate, even though an increased association was obtained. Myo-inositol is a glucose naturally found in the body, and its existence is necessary for the signaling of different molecules, such as insulin, to help its proper functioning. The presence of high amounts of myo-inositol in the follicular fluid indicates good and favorable oocyte quality. The amount of myo-inositol in the body of most women who suffer from PCOS is lower than normal, and therefore, receiving inositol can improve the substance level in the body of this group of women and will help them to increase the oocyte quality (1, 2, 35–37).

Our results showed that considering the age of women with PCOS, with increasing age, the effect of myo-inositol/D-chiro-inositol on clinical pregnancy rate increases. Additionally, as the BMI increases, the impact of this medication on the clinical pregnancy rate also grows. Yet, the influence of age and BMI was not deemed statistically significant in the meta-regression analysis.

Research showed that the myo-inositol level in the follicular fluid of the oocytes of older women was lower than expected, which could contribute to their low oocyte quality (38, 39). By providing sufficient amounts of myo-inositol and folic acid, inositol increases the quality of oocytes resulting in a successful pregnancy (39). On the one hand, inositol solves the insulin resistance problem in these women, in contrast it reduces the levels of estrogen and testosterone to create a hormonal balance in the body. By improving the quality of oocytes, increasing the number of mature oocytes, balancing the level of hormones, and reducing the severity of PCOS, the chances of ovulation and, consequently, pregnancy increase in women consuming myo-inositol (28, 40–42).

The results of the present meta-analysis showed that women with PCOS consuming myo-inositol/D-chiro-inositol had a 17% higher likelihood of increasing the number of top-grade embryos compared to those in the placebo group. This association was statistically significant, with a narrow confidence interval (1.08–1.28), indicating a consistent effect across the studies. Therefore, women in the intervention group were at least 8% and at most 28% more likely to produce top-grade embryos. The improvement in embryo quality can be attributed to the effects of myo-inositol, which has been shown to modify gene expression in granulosa cells and enhance the quality of oocytes and embryos in women with PCOS undergoing ART. These findings further support the potential of myo-inositol as a valuable supplement in improving ART outcomes for women with PCOS (3).

A sufficient and appropriate number of studies were included in the present meta-analysis to assess the mean number of metaphase II oocytes retrieved after the intervention in both groups consuming myo-inositol/D-chiro-inositol and placebo. The combined results indicated that, in women with PCOS, those consuming myo-inositol/D-chiro-inositol had a higher mean number of metaphase II oocytes retrieved compared to the placebo group. However, this difference was not statistically significant. A key limitation of this association was the high heterogeneity observed among the studies, which could be attributed to variations in sample size, age, and BMI of the studied women. The results of the meta-regression analysis further supported this, showing that the mean number of metaphase II oocytes retrieved significantly decreased with increasing age and BMI.

OHSS, which occurs after taking ovulation-stimulating drugs, is a serious and dangerous complication that can even threaten a person's life in some cases (20, 38). In the current meta-analysis, the results showed that in the myo-inositol user group, the probability of OHSS was 16% lower than that of the placebo group. This finding is consistent with the other results of this article that the amount of AFC and AMH decreases in the recipients of myo-inositol. Also, our meta-analysis demonstrated that with increasing age and BMI, the effect of myo-inositol/D-chiro-inositol on OHSS rate decreases, but not significantly.

While the recent publication referenced provides a comprehensive review of the efficacy of myo-inositol and D-chiro-inositol in the management of PCOS, this meta-analysis offers several key contributions that differentiate it from existing literature (43). One major distinction is the focus on ART outcomes, which has not been extensively explored in previous reviews. This analysis specifically addresses ART-related measures such as clinical pregnancy rates, top-grade embryos, and ovarian function markers (AFC and AMH), which are highly relevant to clinical decision-making in women with PCOS undergoing fertility treatments. By narrowing the focus to ART outcomes, this meta-analysis provides more precise insights into how myo-inositol and D-chiro-inositol may influence fertility treatment success, offering valuable information for clinicians involved in the management of ART cycles in PCOS patients. Furthermore, in contrast to studies that have included non-randomized and before-and-after studies, this meta-analysis restricted the analysis to randomized clinical trials, ensuring the highest level of evidence and reducing the risk of bias. This methodological rigor strengthens the reliability of the findings, offering a clearer understanding of the effects of myo-inositol and D-chiro-inositol on ART outcomes. The study also includes an updated and thorough literature search, incorporating data published up to January 2023, ensuring that the conclusions reflect the most current evidence. This makes the meta-analysis a timely and relevant contribution to the field, as it addresses gaps left by previous reviews and provides clinicians with robust, evidence-based recommendations for incorporating myo-inositol and D-chiro-inositol in ART treatment protocols for women with PCOS.

One of the key limitations of the present meta-analysis was the small number of clinical trials that accounted for important and potentially impactful variables, such as prescribed dosages, frequency of doses per day, age, and BMI of women with PCOS. This lack of detailed reporting hindered our ability to perform subgroup analyses and identify the primary sources of heterogeneity across the studies. It is noteworthy that the following article has recently been retracted: Papaleo et al. (18). However, the reason for its retraction was not related to methodological concerns; therefore, the study was not excluded from this review. Moreover, the retraction occurred after the completion of data analysis and manuscript preparation.

## 5. Conclusion

According to the findings of this current meta-analysis, the consumption of myo-inositol/D-chiro-inositol has a notable impact on the clinical pregnancy rate, boosting the number of high-quality embryos while reducing the average AFC and average AMH levels in women suffering from PCOS. The current findings offer adequate and authentic data and proof of the impact of myo-inositol/D-chiro-inositol on fertility and ovarian function outcomes in PCOS women prior to and during ART treatment.

##  Data Availability

Data and materials are available within the complementary materials, and further information can be available by request to the corresponding author.

##  Author Contributions

Y. Moradi and A. Akbari Sene: Concept development (provided idea for the research). Y. Moradi, A. Akbari Sene, M. Saeedzarandi, and M. Yazdizadeh: Search strategy. SR. Ghaffari and F. Amjadi: Data extraction. Y. Moradi: Supervision. Z. Zandieh, M. Saeedzarandi, and A. Akbari Sene: Analysis/interpretation. All authors: Writing (responsible for writing a substantive part of the manuscript).

This manuscript represents a highly collaborative effort between researchers with complementary expertise. M. Saeedzarandi led the clinical and conceptual development of the study, while Y. Moradi oversaw the methodological design, statistical analysis, and interpretation of results. Given the interdisciplinary nature of the research-spanning both clinical practice and advanced meta-analytic techniques-designating both authors as corresponding ensures timely, accurate, and comprehensive communication with the journal and readers. This shared responsibility reflects their equal leadership roles and facilitates broader engagement across different research and clinical communities.

##  Conflict of Interest

The authors declare that there is no conflict of interest.
